# Suicide prevention plans and suicide mortality in Spain, 2010-2023: an interrupted time-series analysis

**DOI:** 10.3389/fpsyt.2025.1709920

**Published:** 2025-12-08

**Authors:** Gerardo Flórez, Ashkan Espandian, Teresa Seoane-Pillado, José Manuel Gerpe, Rocío Villa, Antonio Serrano-García, Pilar Saiz

**Affiliations:** 1 Addiction Treatment Unit, Ourense University Hospital, Ourense, Spain; 2 Center for Biomedical Research in Mental Health Network (CIBERSAM), Madrid, Spain; 3 Psychiatry Service, León Hospital, León, Spain; 4 Area of Preventive Medicine and Public Health, Department of Health Sciences, University of A Coruña-INIBIC, A Coruña, Spain; 5 Demographic Surveys Section, Spanish Office for National Statistics, Ourense, Spain; 6 Health Service of the Principality of Asturias (SESPA), Oviedo, Spain; 7 Health Research Institute of the Principality of Asturias (ISPA), Oviedo, Spain; 8 University Institute of Neurosciences of the Principality of Asturias (INEUROPA), Oviedo, Spain; 9 Department of Psychiatry, University of Oviedo, Oviedo, Spain

**Keywords:** prevention, suicide, interrupted time series analysis, strategies, effectiveness

## Abstract

**Background:**

The serious problem posed by suicide deaths has led to increased awareness and the implementation of prevention plans in a large number of countries worldwide. The main aim of this study is to determine the relationship between the variation in completed suicide rates in Spain and its autonomous communities from 2010 to 2023, and the implementation of suicide prevention strategies at an autonomous community level.

**Methods:**

The incidence rates of suicide deaths in the nineteen Spanish autonomous communities from 2010 to 2023 were obtained from the Spanish Statistical Office. Suicide prevention strategies were classified using the Universal–Selective-Indicated model, with strategies including interventions at all three levels considered multilevel. An interrupted time series analysis was conducted to evaluate the impact of suicide prevention programs on suicide rates. The fitted model included the pre-intervention trend, the immediate change after implementation, and the change in slope from that point onward. Suicide rates at the national level and in Murcia, where no prevention plan was implemented, were included as control variables.

**Results:**

None of the tested interrupted time series analysis variants showed a significant reduction in the rate of completed suicides in any autonomous community. The only exceptions were the first year of implementation of the prevention plan in Galicia and Madrid after adjusting for the national rate and using Murcia as the control region for the after-intervention analysis.

**Conclusion:**

Despite the suicide prevention plans launched by numerous Spanish autonomous communities, to date no significant reduction in the evolution of completed suicide rates has been demonstrated in any of these autonomous communities or nationwide.

## Introduction

Suicide is a serious public health problem worldwide and is one of the leading causes of preventable death (1).This situation leads to an urgent need to implement and improve prevention strategies in all countries around the world (2).

According to data provided by the World Health Organization (WHO), the number of suicide deaths reaches nearly 700,000 people per year. The global rate is estimated at 9.4 suicides per 100,000 inhabitants, with a higher percentage among males. It represents the fourth leading cause of death for the young adult population (15-29 years) worldwide (3). According to data from the National Institute of Statistics (INE) (4), in 2022, Spain recorded the highest number of suicide deaths in its history, exceeding those of 2021 by 5.6 percent. The latest data published for 2023 show a slight decrease of 2.6 percent, highlighting the possibility of the beginning of a downward trend (4).

Given the challenge that these high suicide death rates pose to population health, institutions such as the World Health Organization (WHO) and the International Association for Suicide Prevention (IASP) have developed common guidelines, which include the following recommendations for establishing suicide prevention strategies (3, 5): preventive measures should address suicide and suicide attempts; providing support for people at risk can prevent suicide and should be implemented; national governments must develop strategies that provide financial and technical support for prevention strategies involving society as a whole; and finally, measurable objectives and systematic studies should be presented.

Among the prevention strategies currently implemented, only a few have sufficient evidence to demonstrate their effectiveness in reducing the incidence of suicide (6): general practitioner education, youth suicide prevention education, pharmacotherapy, psychotherapy (cognitive behavioral therapy, dialectical behavioral therapy and psychodynamic therapies), contact and active outreach, and firearms restriction.

It remains unclear to date which strategy is most effective, and no comparative estimate of the effect of different types of interventions has been provided (7). Work continues in the hope of identifying the key to suicide prevention (8). Therefore, it is currently suggested that an effective plan to reduce suicide would require combined interventions by different professionals in multiple areas, known as multilevel interventions (8, 9). These multilevel interventions may have synergistic potential, meaning that the effect of the combined parts of the intervention could create a greater impact than the sum of the individual effects of the interventions (10). An example of this is the prevention strategy implemented in the diathesis model. This has shown favorable results in a combined intervention at different stages of action (6): awareness and education, screening for individuals at high risk, treatment, follow-up care for suicide attempts, and means restriction.

These interventions can be classified according to the Universal-Selective-Indicated (USI) model, which defines three levels of prevention (1). Universal prevention: strategy aimed at the general population (nations, states, communities, schools); selective prevention: for subgroups of people with risk factors for suicidal behavior (biological or psychosocial) even if they do not currently exhibit suicidal thoughts or behaviors; and indicated prevention: for individuals in the population who have risk factors or conditions that place them at high risk.

For all the above reasons, and considering the tools currently available, many countries have implemented prevention plans. Currently, more than 45 countries have a prevention plan in place, notably Finland and South Korea, both of which have been able to demonstrate a reduction in suicide mortality following the implementation of their plans (11, 12).

In Spain, health authorities are aware of the seriousness of suicidal behavior, and therefore various suicide prevention plans have been developed at the regional level in most of the Spanish autonomous communities, although their degree of implementation (13)varies. The priority given to this issue has led to the development of a comprehensive national suicide prevention and treatment plan of suicidal behavior, scheduled to begin in 2025 (14).

The main aim of this study is to determine the relationship between the variation in suicide mortality rates in Spain and its autonomous communities from 2010 to 2023 and the implementation of suicide prevention strategies at an autonomous community level.

The working hypothesis is that the implementation of suicide prevention strategies has a significant influence on the variation of suicide mortality rates in Spain and its autonomous communities from 2010 to 2023. Specifically, suicide rates are expected to show a significant decrease. If this hypothesis is verified, the importance of implementing these suicide prevention strategies at a national level would be strongly supported.

## Material and methods

### Data

The incidence rates of suicide deaths for the nineteen Spanish autonomic regions from 2010 to 2023 were obtained from the Spanish Statistical Office (INE) (https://www.ine.es).

The prevention plans implemented in the different autonomous communities were obtained from the following websites:

Andalusia: https://www.sspa.juntadeandalucia.es/servicioandaluzdesalud/sites/default/files/sincfiles/wsas-media-mediafile_sasdocumento/2023/programa_prevencion_conductas_suicidas_andalucia_2023_2026_def.pdf

Aragón: https://www.aeesme.org/wp-content/uploads/2022/11/ESTRATEGIA-DE-PREVENCION-DEL-SUICIDIO-EN-ARAGON.pdf

Asturias: https://cendocps.carm.es/documentacion/2019_Protocolo_deteccion_manejo_caso_suicidio.pdf

Balearic Islands: https://www.ibsalut.es/es/servicio-de-salud/organizacion/coordinaciones-autonomicas-sanitarias/coordinacion-autonomica-de-salud-mental-de-las-islas-BalearicIslands/4031-plan-de-prevencion-actuacion-y-abordaje-de-la-conducta-suicida-en-las-islas-BalearicIslands.

Canary Islands: https://www3.gobiernodecanarias.org/sanidad/scs/contenidoGenerico.jsp?idDocument=8745abde-0fff-11ec-8489-0f64eb00dcc8&idCarpeta=28bb0a80-11f9-11ef-9021-37a5c312dc04

Cantabria:

https://www.scsalud.es/plan-de-salud-mental

Castile and Leon: https://www.saludcastillayleon.es/institucion/es/planes-estrategias/estrategia-prevencion-conducta-suicida-castilla-leon-2021-2.ficheros/2140352-Estrategia%20de%20prevenci%C3%B3n%20de%20la%20conducta%20suicida%20en%20Castilla%20y%20Le%C3%B3n%202021-2025.pdf

Castile-La Mancha: https://sanidad.castillalamancha.es/sites/sescam.castillalamancha.es/files/documentos/pdf/20201123/guia_preven_suic_ijv.pdf

Catalonia: https://scientiasalut.gencat.cat/bitstream/handle/11351/6319/pla_prevencio_suicidi_catalunya_2021_2025_2021.pdf?isAllowed=y&sequence=4

Extremadura:

https://saludextremadura.ses.es/filescms/smex/uploaded_files/CustomContentResources/II%20PLAN%20DE%20ACCION%20PARA%20LA%20PREVENCION%20Y%20ABORDAJE%20DE%20LAS%20CONDUCTAS%20SUICIDAS%20EN%20EXTREMADURA.pdf

Galicia: https://www.aeesme.org/wp-content/uploads/2022/11/plan-de-prevencion-del-suicidio-en-galicia.pdf

Madrid: https://www.comunidad.madrid/transparencia/informacion-institucional/planes-programas/plan-prevencion-del-suicidio-comunidad-madrid-2022-2026

Navarre: https://www.navarra.es/NR/rdonlyres/6683F5F2-AFA8-43DB-B090-7B42CEAE3BC5/478565/PlandeatencionalaspersonasconconductassucidasenlaR.pdf

Basque Country: https://www.euskadi.eus/contenidos/informacion/estrategia_prevencion_suicidio/es_def/adjuntos/plan_prevencion_suicidio_cast.pdf

La Rioja: https://www.riojasalud.es/files/content/ciudadanos/planes-estrategicos/PLAN_PREVENCION_CONDUCTA_SUICIDA_DEF.pdf

Valencian Community: https://www.san.gva.es/es/web/assistencia-sanitaria/pla-prevencio-suicidi

To classify the suicide prevention strategies of the autonomous communities, the Universal- Selective-Indicated (USI) model was used. Universal interventions are designed to influence an entire population. Selective interventions are designed to focus on at-risk groups with greater probability of becoming suicidal. Indicated interventions address individuals showing signs of suicidal risk. Strategies that included interventions from all three levels of the USI were considered multilevel.

### Ethics aspects

The authors complied with all the contents set out in the current legislation on clinical research established in the Declaration of Helsinki, European Convention on Human Rights and Biomedicine, and the UNESCO Universal Declaration on Human Rights. They complied with the requirements established under Spanish legislation in the field of medical research, personal data protection and bioethics, as well as all other requirements set out by Spanish legislation on this topic. Written informed consent for participation was not required for this study in accordance with national legislation and institutional requirements. The databases used are fully anonymized, and their use raises no ethical concern. In addition, they are government-owned and accessible to all citizens. The current research did not involve human or animal studies.

### Statistical analysis

An interrupted time series analysis (ITS) was conducted to evaluate the impact of suicide prevention programs on suicide rates across Spain’s autonomous communities. This methodology allows modeling the behavior of a variable over time, explicitly incorporating the timing of an intervention to assess whether it results in a significant change in the level or trend of the time series.

An ordinary linear interrupted time series model was used, since the dependent variable corresponds to continuous rates (range: 1.16-14.03 per 100,000 inhabitants-year).

The fitted model included the pre-intervention trend, the immediate change following implementation, and the change in slope from that point onward, when data availability allowed. In addition, the national suicide rate and the Murcia suicide rate, where no prevention plan was implemented, were included as control variables to account for fluctuations common to all regions.

All analyses were performed using R software (version 4.4.1), and the packages tidy verse, ggplot2, and lmtest were used. A p-value < 0.05 was considered statistically significant.

## Results

All suicide prevention strategies were multilevel, except those implemented in Asturias and Castile-La Mancha.

**Table 1** shows the prevalence of suicide deaths in Spain and its autonomous communities from 2010 to 2023; it also indicates the year in which each suicide prevention plan was initiated in each autonomous community.

**Table 1:** Prevalence of suicide deaths in Spain and its autonomous communities between 2010 and 2023.

Autonomous communities	2010	2011	2012	2013	2014	2015	2016	2017	2018	2019	2020	2021	2022	2023
Spain	**6.72**	**6,74**	**7,49**	**8,21**	**8.36**	**7.73**	**7.67**	**7.90**	**7.57**	**7.81**	**8.31**	**8.45**	**8.63**	**8.51**
Andalusia	8.18	7.73	9.24	9.57	9.33	8.12	7.96	8.26	7.79	7.61	9.37	10.02	9.58	9.49
Aragón	7.05	6.61	7.34	7.27	8.75	9.94	8.64	7.64	9.17	8.26	7.67	8.60	8.90	7.75
Asturias	13.74	11.10	12.25	13.86	14.03	12.84	12.85	12.95	13.42	12.51	11.98	12.85	12.14	13.92
Balearic Islands	8.68	7.82	8.04	9.36	8.79	8.42	8.31	9.23	6.29	8.44	7.43	7.59	9.09	7.19
Basque Country	5.78	7,87	8.21	8.26	8.50	7.67	8.18	8.29	7.41	6.21	8,06	6.64	7.25	6.36
Canary Islands	7.36	6.68	8.92	8.73	8.46	9.05	8.71	9.49	9.07	9.15	9.56	10.58	10.65	10.89
Cantabria	3.38	6.24	6.06	5.41	6.46	6.49	7.04	4.65	4.83	6.20	7.89	8.21	10.42	7.82
Castile and Leon	8.44	7.11	7.86	8.33	9.62	9.06	8.78	8.99	8.84	9.09	9.52	9.90	10.37	8.39
Castile-La Mancha	6.72	6.85	7.68	8.6	9.14	8.11	6.61	7.73	6.51	7.48	8.80	8.25	8.86	7.68
Catalonia	5.56	6.34	6.95	7.20	7.06	6.59	6.78	6.67	6.87	6.97	7.15	7.42	7.79	7.99
Extremadura	7.32	6.31	6.86	6.70	5.73	5.31	7.54	7.13	6.52	7.40	8.65	7.65	8.82	7.87
Galicia	9.83	10.95	11.94	12.04	13.50	11.64	12.43	11.93	10.14	10.82	11.29	12.50	11.49	11.37
Madrid	1.89	2.14	1.46	5.13	5.35	5.08	4.72	5.24	5.20	5.25	5.50	5.15	5.70	6.08
Murcia	6.16	7.89	7.19	6.93	7.23	7.22	8.12	6.67	8.59	8.37	8.07	8.10	8.75	7.60
Navarre	6.12	7.94	7.45	8.07	7.33	8.28	7.96	7.46	6.02	7.03	6.65	9.07	8.73	9.67
Rioja	5.27	7.12	9.27	7.45	9.72	7.89	8.87	7.93	6.65	9.15	9.06	7.50	9.38	9.31
Valencian Community	7.83	6.49	7.92	7.80	7.59	6.99	6.77	8,03	7.98	8.99	8.70	7.97	8.55	8.97
Ceuta	4.96	1.21	3.57	4.75	7.06	3.56	4.73	3.53	1.17	4.72	4.75	4.79	2.41	6.02
Melilla	1.32	6.37	7.43	1.20	5.92	4.67	2.32	2.32	1.16	1.16	4.59	4.64	1.17	7.02

In blue the year in which the suicide prevention plan was initiated in each autonomous community, in grey the years after the start of the plan.Significant p-values in bold.

**Table 2** shows the Interrupted Time Series (ITS) regression for each autonomous community. According to the model fit some provinces exhibited significant pre-existing trends (Beta Time). While the ITS model statistically accounts for these trajectories, they remain relevant for interpreting the intervention’s impact, as perceived effectiveness can differ depending on whether the province was already experiencing an upward or downward trajectory before implementation.

**Table 2:** Interrupted Time Series (ITS) Regression for each autonomous community.

Autonomous communities	Intercept	Beta time	*P* time	Beta intervention	*P* intervention	Beta post	*P* post	R2
Andalusia	8.252	0.074	0.200	0.534	0.509	NA	NA	0.133
Aragón	7.097	0.216	**0.045**	-1.106	0.259	-0.161	0.691	0.350
Asturias	1.737	0.062	0.685	-0.586	0.577	0.002	0.994	0.032
Balearic Islands	8.715	-0.094	0.198	1.505	0.146	-1.809	0.148	0.317
Basque Country	7.322	0.119	0.295	-1.388	0.147	-0.169	0.565	0.332
Canary Islands	7.603	0.210	**0.002**	0.647	0.312	-0.057	0.886	0.835
Cantabria	4.893	0.214	0.055	2.957	0.059	-2.816	0.123	0.650
Castile and Leon	7.935	0.152	**0.027**	0.704	0.331	-0.908	0.066	0.589
Castile-La Mancha	7.636	-0.017	0.866	1.344	0.194	-0.258	0.545	0.231
Catalonia	6.338	0.080	0.052	0.225	0.609	0.203	0.475	0.686
Extremadura	6.644	-0.009	0.941	0.535	0.544	0.295	0.224	0.501
Galicia	10.608	0.384	**0.036**	-2.225	**0.034**	-0.287	0.228	0.414
Madrid	2.566	0.323	**0.004**	-0.739	0.561	0.056	0.970	0.636
Murcia	6.880	0.116	**0.014**	NA	NA	NA	NA	0.405
Navarre	7.568	-0.053	0.485	1.875	0.054	0.353	0.534	0.593
Rioja	6.880	0.303	0.133	-1.675	0.218	0.052	0.883	0.333
Valencian Community	7.606	-0.088	0.393	1.155	**0.070**	0.193	0.198	0.639
Ceuta	3.836	0.039	0.739	NA	NA	NA	NA	0.010
Melilla	3.930	-0.041	0.807	NA	NA	NA	NA	0.005

Beta Time, measures the slope of the rate before the intervention; that is, the change per year during the pre-phase. A positive value indicates that the rate was increasing before the intervention. A negative value suggests that the rate was decreasing annually. The *p*-value indicates whether the pre-phase slope is statistically different from 0.

Beta Intervention, reflects the immediate change in the rate in the autonomous communities when the intervention is implemented. A positive value indicates an increase in the rate right in the start year, while a negative value implies a decrease. The associated p-value indicates whether this change is statistically significant.

Beta Post, indicates the change in slope after the intervention was initiated compared to the previous slope. A positive value means that, after the intervention, the rate is increasing faster each year than before. A negative value suggests that the post-intervention trend is more downward than the pre-phase. The *p*-value indicates whether the change in slope is statistically significant.

R2, The closer to 1, the better the fit; lower values indicate that the model explains less of the observed variation.

NA, not applicable.

Significant p-values in bold.

**Table 3** shows the Interrupted Time Series (ITS) regression for each autonomous community, adjusting for the national suicide rate. The Canary Islands, the Balearic Islands and Castile-La Mancha showed significant pre-existing trends that may have influenced the intervention’s impact (Beta time).

**Table 3:** Interrupted Time Series (ITS) regression for each autonomous community adjusting for the national suicide rate.

Autonomous communities	Intercept	Beta time	*P* time	Beta int	*P* int	Beta post	*P* post	Beta national	*P* national	R2
Andalusia	-2.718	-0.112	0.104	0.528	0.446	NA	NA	1.544	**0.004**	0.649
Aragón	3.719	0.172	0.162	-1.209	0.237	-0.155	0.710	0.469	0.474	0.387
Asturias	6.652	-0.080	0.681	-0.025	0.982	-0.036	0.896	0.866	0.273	0.160
Balearic Islands	1.738	-0.199	**0.040**	1.324	0.158	-1.589	0.160	0.975	0.094	0.508
Basque Country	-0.933	-0.012	0.913	-1.192	0.143	-0.239	0.343	1.155	**0.046**	0.582
Canary Islands	2.462	0.137	**0.036**	0.498	0.368	-0.007	0.983	0.717	0.060	0.891
Cantabria	-1.269	0121	0.388	2.797	0.075	-2.622	0.153	0.861	0.333	0.687
Castile and Leon	2.820	0080	0.264	0.555	0.401	-0.859	0.060	0.713	0.108	0.696
Castile-La Mancha	-5.600	-0.189	**0.005**	0.940	0.061	-0.234	0.239	1.839	**0.001**	0.860
Catalonia	1.579	0.013	0.682	0.087	0.771	0.249	0.212	0.663	**0.006**	0.871
Extremadura	8.680	0.038	0.825	0.347	0.733	0.308	0.230	-0.290	0.670	0.511
Galicia	-0091	0.071	0.649	-1.155	0.152	-0.226	0.198	1.540	**0.011**	0.725
Madrid	-7.410	0.173	0.121	-0.998	0.367	0.370	0.777	1.395	0.056	0.763
Murcia	10.102	0.166	**0.023**	NA	NA	NA	NA	-0.451	0.320	0.459
Navarre	3.038	-0.117	0.227	1.743	0.071	0.397	0.481	0.632	0.273	0.647
Rioja	-1.784	0.100	0.676	-0.877	0.535	-0.003	0.992	1.233	0.208	0.446
Valencian Community	3.273	-0.215	0.111	1.589	**0.026**	0.218	0.132	0.624	0.144	0.719
Ceuta	-12.534	-0.215	0.195	NA	NA	NA	NA	2.292	0.056	0.299
Melilla	-2.274	-0.137	0.614	NA	NA	NA	NA	0.869	0.644	0.025

Beta Time, measures the slope of the rate before the intervention; that is, the change per year during the pre-phase. A positive value indicates that the rate was increasing before the intervention. A negative value suggests that the rate was decreasing annually. The *p*-value indicates whether the pre-phase slope is statistically different from 0 or not. Beta Int (Intervention), reflects the immediate change in the rate in the autonomous communities when the intervention is implemented. A positive value indicates an increase in the rate right in the start year, while a negative value implies a decrease. The associated pvalue indicates whether this change is statistically significant or not.

Beta Post, indicates the change in slope after the intervention compared to the previous slope. A positive value means that, after the intervention, the rate is increasing faster each year than before. A negative value suggests that the post-intervention trend is more downward than the pre-phase. The *p*-value indicates whether the change in slope is statistically significant or not.

Beta National, Measures the influence of the national suicide rate evolution on the variation of the suicide rate in each autonomous community. The p-value indicates whether this influence is statistically significant or not.

R2, The closer to 1, the better the fit; lower values indicate that the model explains less of the observed variation.

NA, not applicable.

Significant p-values in bold.

Valencian Community, Cantabria and Navarre showed significant or near-significant increases after the intervention (Beta intervention).

Castile and Leon showed a near-significant decrease in the post-intervention trend (Beta post).

Andalusia, Castile-La Mancha, Galicia, Catalonia, and the Basque Country showed a strong influence from the national suicide rate. This strong synchrony between the evolution of these regions’ rates and the national rate may reflect both the influence of national factors and the impact that events in large-population regions (Andalusia and Catalonia) exert on the national aggregate.

**Table 4** shows the Interrupted Time Series (ITS) analysis, adjusting for the national suicide rate and considering Murcia as a control community, as no intervention was implemented there. The results from the pre-intervention trend show that Aragon, the Canary Islands, Madrid, and Murcia showed significant upward trends in suicide rates before the intervention (Beta time). Therefore, in these regions, suicide rates were already increasing regardless of the program.

**Table 4:** Interrupted Time Series (ITS) analysis adjusting for the national suicide rate and considering Murcia as a control community, given that no intervention was implemented in this region.

Autonomous communities	Intercept	Beta time	*P* time	Beta int	*P* int	Beta post	*P* post	Beta national	*P* national	Beta murcia	*P* murcia	Beta Int mod	*P* int mod	R2
Andalusia	4.416	0.014	0.817	0.517	0.658	NA	NA	0.537	0.143	-1.372	**0.012**	0.042	0.530	0.548
Aragón	7.071	0.215	**0.026**	-1.107	0.189	-0.161	0.645	0.004	0.992	-0.216	0.714	-0.100	0.314	0.421
Asturias	11.767	0.039	0.787	-0.497	0.586	-0.004	0.986	0.138	0.748	-5.872	**0.001**	0.062	0.657	0.935
Balearic Islands	6.881	-0.121	0.105	1.458	0.100	-1.751	0.101	0.256	0.487	-1.831	**0.003**	0.209	**0.013**	0.441
Basque Country	4.869	0.080	0.442	-1.330	0.099	-0.190	0.452	0.343	0.357	-0.441	0.452	-0.002	0.985	0.395
Canary Islands	6.687	0.197	**0.006**	0.620	0.362	-0.048	0.910	0.128	0.674	-0.719	0.125	-0.095	0.183	0.817
Cantabria	3.464	0.193	0.051	2.920	**0.015**	-2.771	**0.049**	0.200	0.675	1.989	**0.009**	-0.099	0.332	0.675
Castile and Leon	7.032	0.139	0.052	0.677	0.346	-0.900	0.057	0.126	0.694	-1.050	**0.038**	-0.037	0.615	0.702
Castile-La Mancha	2.740	-0.081	0.356	1.194	0.138	-0.249	0.457	0.680	0.073	-0.718	0.209	0.121	0.201	0.412
Catalonia	5.612	0.070	0.235	0.204	0.735	0.210	0.586	0.101	0.709	0.546	0.190	0.035	0.580	0.618
Extremadura	9.306	0.053	0.675	0.289	0.717	0.312	0.135	-0.379	0.319	0.280	0.633	0.105	0.389	0.532
Galicia	7.634	0.297	0.071	-1.928	**0.029**	-0.270	0.175	0.428	0.275	-3.810	**0.001**	-0.228	0.138	0.915
Madrid	-0.755	0.273	**0.003**	-0.825	0.410	0.161	0.893	0.464	0.281	4.320	**0.001**	-0.209	**0.029**	0.859
Murcia	10.102	0.166	**0.023**	NA	NA	NA	NA	-0.451	0.320	NA	NA	NA	NA	0.459
Navarre	6.955	-0.062	0.425	1.857	**0.029**	0.359	0.482	0.085	0.812	-0.685	0.213	0.168	0.052	0.531
Rioja	4.757	0.253	0.143	-1.480	0.173	0.038	0.888	0.302	0.549	-0.035	0.964	-0.170	0.299	0.406
Valencian Community	7.443	-0.093	0.474	1.172	0.097	0.194	0.231	0.023	0.941	-0.730	0.161	0.207	0.103	0.538
Ceuta	-2.738	-0.063	0.570	NA	NA	NA	NA	0.920	0.161	3.045	**0.003**	0.077	0.522	0.721
Melilla	2.438	-0.064	0.692	NA	NA	NA	NA	0.209	0.824	2.951	**0.036**	0.157	0.374	0.592

Beta Time, measures the slope of the rate before the intervention; that is, the change per year during the pre-phase. A positive value indicates that the rate was increasing before the intervention. A negative value suggests that the rate was decreasing annually. The *p*-value indicates whether the pre-phase slope is statistically different from 0 or not. Beta Int (Intervention), reflects the immediate change in the rate in the autonomous communities when the intervention is implemented. A positive value indicates an increase in the rate right in the start year, while a negative value implies a decrease. The associated pvalue indicates whether this change is statistically significant or not.

Beta Post, indicates the change in slope after the intervention compared to the previous slope. A positive value means that, after the intervention, the rate is increasing faster each year than before. A negative value suggests that the post-intervention trend is more downward than the pre-phase. The *p*-value indicates whether the change in slope is statistically significant or not.

Beta National, measures the influence of the national suicide rate evolution on the variation of the suicide rate in each autonomous community. The p-value indicates whether this influence is statistically significant or not.

Beta Murcia, uses Murcia as a control community with respect to the evolution of suicide rates after the intervention in the rest of the autonomous communities. The associated p-value indicates whether this comparison is statistically significant or not.

Beta Int (intervention) mod, represents the change in the slope of the suicide rate after the intervention, adjusting for the national rate and using Murcia as a control región. The associated p-value indicates whether this change is statistically significant or not.

R2, The closer to 1, the better the fit; lower values indicate that the model explains less of the observed variation.

NA, not applicable.

Significant p-values in bold.

Regarding the immediate effect after the intervention was initiated (Beta intervention), Galicia experienced an immediate and significant reduction in the suicide rate. The Basque Country showed a reduction that approached statistical significance. However, Cantabria showed the opposite effect, with a significant increase immediately after the intervention, similar to Navarre, although in this case the effect was not statistically significant.

The change in the post-intervention trend showed the following: Cantabria and Castile and Leon showed reductions in the post-intervention trend. Therefore, although Cantabria experienced an immediate increase, the long-term trend shows a significant decrease in the suicide rate. Castile and Leon also showed a reduction close to the threshold of statistical significance. The Balearic Islands and Galicia recorded decreases in the post-intervention trend, although these did not reach statistical significance.

When adjusted for the national rate (Beta national), Castilla-La Mancha and Madrid followed a similar trend to the national rate, showing relatively strong relationships, although they did not reach statistical significance. The rest of the autonomous communities did not show a significant relationship with the national rate.

Using Murcia as a control group (Beta Murcia), Asturias, Galicia, Castile and Leon and the Balearic Islands showed significantly lower suicide rates than Murcia following the intervention. However, Madrid and Cantabria showed significantly higher rates than Murcia. No significant differences were observed in the remaining autonomous communities.

The Beta_Intervention model represents the change in the post-intervention trend of the suicide rate, adjusted for the national rate and considering Murcia as the control region. Therefore, it measures whether the intervention had a differential impact on the trend in each autonomous community, compared to the expected evolution at the national level and in Murcia. For Madrid, the change in slope was statistically significant; so, after adjustment, the intervention appeared to reduce the increase in the suicide rate. For the Balearic Islands, although the absolute change indicated a reduction in the slope, the adjusted analysis showed a significant increase in the trend compared to Murcia. No significant differences were shown in the remaining autonomous communities.

## Discussion

The results of this study yield a clear conclusion. None of the ITS variants tested showed a significant reduction in completed suicide rates in any autonomous community. The only exceptions were the first year of implementation of the prevention plan in Galicia (**Tables 2** and **Table 4**) and in Madrid after adjusting for the national rate and considering Murcia as the control region for the post-intervention analysis (**Table 4**). Therefore, none of the prevention plans studied were able to demonstrate with a robust statistical significance their ability to reduce completed suicide rates in Spain.

However, in Aragon, the Canary Islands, Cantabria, Castile and Leon, and Galicia, a consistent, though not statistically significant, decrease in completed suicide rates was observed across all the ITS models tested (**Tables 2**–**4**). This finding is relevant since these rates were increasing in these autonomous communities prior to the intervention (**Tables 2**–**4**).

It would also be important to note the significant findings for Cantabria (**Table 4**) and the Balearic Islands (**Table 4**), which, although counterintuitive, could suggest a deleterious effect—an increase in suicide rates. While it may seem implausible to infer a causal relationship between a policy designed to reduce suicide rates and an observed increase, such a scenario is not impossible. For instance, Damiano et al. (2024) (15) associated an increase in suicide rates in Brazil (2000–2019) with the launch of a nationwide suicide awareness media campaign in September 2015. Reporting such results could guide policymakers to examine whether potentially problematic aspects of these regional plans might require correction or improvement.

Therefore, the results of this study indicate a promising line of work, but one that has not yet demonstrated sufficient effectiveness to achieve the desired effect: a significant reduction in completed suicide rates in the Spanish autonomous communities or nationwide.

What has caused this lack of effectiveness and how to correct it? What lessons can be learned from previous experiences in countries such as Estonia, Scotland, Japan, and Korea that have been able to reduce their suicide rates? (11, 12, 16, 17).

The first aspect to be noted is that the variable that marks the success or failure of these plans-the reduction in suicide death rates-and that has been used in the current study, is difficult to measure. This is due to the following reasons: relative statistical rarity of suicide, naturally occurring fluctuations in suicide rates over time, regression to the mean, and delays between suicide death registration and publication of mortality data (18). In order to overcome these problems, time and statistical power is required. These factors have been lacking in some autonomous communities where plans were launched very recently or in small populations. It is essential to consider that the population distribution in the Spanish autonomous communities varies considerably. For example, in the most populated autonomous communities, prevention plans were launched very recently—Madrid in 2021 and Catalonia in 2022—whereas in much less populated regions, plans have been in place for much longer—Asturias, La Rioja, and Extremadura in 2018, and Galicia in 2017.

Another area for improvement is enhancing public suicide awareness to reduce the stigma that surrounds suicide and to promote the perception that suicide is preventable (12).

An exhaustive and ongoing psychological autopsy study is also highly recommended in order to identify key target groups for specific interventions and to verify the effectiveness of these interventions (12). In addition to that, comprehensive and well-designed studies that assess the evolution of suicidal ideation and suicide attempts would also support the evaluation of the effectiveness of such interventions (17, 19).

To achieve a multilevel intervention so that a synergistic potential can be obtained, different providers from multiple domains must be involved. Not only healthcare personnel, but also professionals from the social, educational, and information sectors should also be part of these preventive strategies. This also applies to suicide prevention plans, which should not only be strategies for the health system, but also strategies to restrict access to commonly used means of suicide attempts, alongside programs for public awareness and responsible media reporting (18). Involving stakeholders other than public sector employees is also highly desirable (7). Therefore, integrating social and environmental approaches in addition to individual-centered mental health projects is essential. Achieving this level of preventive excellence is challenging, and it is possible that the suicide prevention plans in the Spanish autonomous communities have not yet reached this standard, as demonstrated by countries such as Korea, which required approximately 10 years to achieve this (11).

Interventions need to cover longer periods of time and target broader and more diverse age groups (16).

The scale of required human and financial resources must be increased (11, 19).

More intense monitoring and evaluation framework is also needed to track progress toward the achievement of strategic objectives (19).

Finally, and as strongly recommended in the international literature (11, 17, 18), it should be necessary to address suicide prevention at the national level. A national plan would allow for the standardization of interventions across the autonomous communities and involve all of them in its implementation, monitoring, and evaluation. It would also demonstrate government’s clear recognition of suicide as a public health priority and that the society is committed to its prevention and reduction (18). For this reason, the Spanish Ministry of Health has launched the 2025-2027 Action Plan for Suicide Prevention. This plan aims to unify and improve the action plans individually implemented by each autonomous community (14). This national plan is designed to ensure that all proposed activities are incorporated and integrated into the everyday performance of health care and social public services.

This study has several limitations. Firstly, suicide prevention plans were implemented in different years in each autonomous community, which complicated the comparison between regions with longer-standing plans and those with more recently established plans. Secondly, data on the implementation of each plan in the respective autonomous community was not available for this study. Therefore, it is not possible for us to determine whether the negative results are attributable to inadequate implementation of the plan or a ineffectiveness of the measures applied. Consequently, it is not clear whether a specific strategy was implemented and, if so, its quality, scale, intensity, completeness and timing (18). In either of the two possible scenarios, negative results would reflect a failure of the suicide prevention plan.

A limitation of this study that has to be acknowledged is that the presence of contextual factors that could significantly and independently affect the evolution of completed suicides in each autonomous community was not taken into account. These demographic, environmental, educational, economic, and health variables, such as the impact of COVID-19, have shown to significantly influence suicide rates (20). It would be important in future studies to control for these contextual factors.

## Conclusions

Unfortunately, despite the suicide prevention plans launched by numerous Spanish autonomous communities, to date no significant reduction in completed suicide rates has been observed either within individual autonomous communities or in the country as a whole. Therefore, the working hypothesis has not been verified. It is necessary to work on a unified national plan that includes all the multilevel interventions that have proven effective in other countries.

## Data Availability

Publicly available datasets were analyzed in this study. This data can be found here: Spanish Office for National Statistics Deaths by cause of death. http://www.ine.es.
